# RLSTM: A New Framework of Stock Prediction by Using Random Noise for Overfitting Prevention

**DOI:** 10.1155/2021/8865816

**Published:** 2021-05-19

**Authors:** Hongying Zheng, Zhiqiang Zhou, Jianyong Chen

**Affiliations:** ^1^School of Software Engineering, Shenzhen Institute of Information Technology, Shenzhen 518172, China; ^2^Guangdong Laboratory of Artificial Intelligence and Digital Economy (SZ), Shenzhen University, Shenzhen 518060, China

## Abstract

An accurate prediction of stock market index is important for investors to reduce financial risk. Although quite a number of deep learning methods have been developed for the stock prediction, some fundamental problems, such as weak generalization ability and overfitting in training, need to be solved. In this paper, a new deep learning model named Random Long Short-Term Memory (RLSTM) is proposed to get a better predicting result. RLSTM includes prediction module, prevention module, and three full connection layers. Input of the prediction module is a stock or an index which needs to be predicted. That of the prevention module is a random number series. With the index of Shanghai Securities Composite Index (SSEC) and Standard & Poor's 500 (S&P500), simulations show that the proposed RLSTM can mitigate the overfitting and outperform others in accuracy of prediction.

## 1. Introduction

Forecasting future price of a financial asset, such as a stock, is essential for investors as it can reduce the risk of decision-making by appropriately determining the future movement of an investment asset. The investors are more likely to buy the stocks whose value is expected to increase in the future. On the other hand, traders are likely to refrain from buying a stock whose value is expected to fall in the future [1]. Therefore, an accurate prediction of stocks market index is very helpful for investors to get more profit. However, with the highly volatile and nonstationary nature of market, it is hard to predict the future price of stock properly [2]. Thus, technical and quantitative methods have been utilized by investors as an attempt to forecast asset price movement. These approaches consist of discovering a suitable pattern from market data and capturing the preferable moment for investment decisions.

Traditional economic models and machine learning algorithms have many practical applications in stock price forecasting [3, 4]. At early stage, the prediction of financial asset price is mainly based on the research of stock behavior [5]. Parametric statistical models, such as Autoregressive Integrated Moving Average (ARIMA) model and Generalized Autoregressive Conditional Heteroskedasticity (GARCH), are used for stock price prediction [6, 7]. Those kinds of models can achieve significant effect in the short-term prediction.

With the development of machine learning, some of them show great capability in stock prediction by virtue of its nonlinear mapping and strong generalization ability. In [8], GARCH is combined with Artificial Neural Networks (ANNs), which is far better than GARCH alone in predicting market volatility in Latin America. Another important machine learning technique, Support Vector Regression (SVR), is a regression model that can show great ability in predicting future data based on historical data. It has many applications in stock forecasting. In [9], a framework is proposed to apply SVR strategy to predict the stock price. In [10], SVR and Hodrick–Prescott filter are combined together in order to get better result in prediction of the stock price. Hou et al. [11] proposed a new method combining SVR and Grey Relation Analysis for short trend stock market prediction which takes advantage of SVR's ability in analyzing small-size and multidimensional samples. The method they proposed has fast convergence and is efficient in stock price prediction. In [12], a framework based on genetic algorithm is proposed which uses both feature selection and support vector machine to optimize time window of the attributes. Experiment results show that the framework can improve the rate of return and get a better prediction performance.

In recent years, deep learning model such as Convolutional Neural Network (CNN) and Long Short-Term Memory Network (LSTM) are increasingly used for the stock price prediction. CNN with its advantage of feature extraction is widely used in the stock price data. A CNN based architecture is proposed to select features in different indicators, price, and temporal information from Borsa Istanbul 100 stocks [13]. It is proved that CNN helps reduce training time and model complexity. In [1], CNN is used to analyze the candlestick chart of a stock market, to find the patterns inside stock market, and to predict the future movements of it. Experiments on dataset of Taiwan and Indonesian stock market have shown a promising result. CNN is also used to build market features in a hierarchical way [14]. With this method, a relationship can be found between fundamental and technical indicators.

LSTM has been extensively utilized for time-series analysis [15–17] which is advantageous over RNN as it overcomes the problem of vanishing gradient. Authors in [18] proved that given the relevant data, LSTM can be used to find the intrinsic trend of stock prices. Chen et al. used historical stock price data of Shanghai Securities Composite Index of China (SSEC) as the input of LSTM to forecast the index [19]. In [20], a stock prediction method based on LSTM is proposed which is used to simulate trading. The experiment results show that the trading strategy has lower risk than that based on other model such as multilayer perceptron and random forest. At the same time, many researchers combined CNN and LSTM together to utilize both advantages in stock price prediction. In [21], a model called TC-LSTM uses CNN to capture the fluctuation features of stock prices and then uses LSTM to predict the future price of the stock. The author in [22] used a kind of combination of CNN and LSTM to analyze quantitative strategy in stock market.

However, these models may meet a problem of weak generalization ability because of two main reasons. First, the stock data appear highly nonlinear. There are many factors that affect the price trend of a stock, including politics and psychology of investors [23]. Moreover, these factors in different time may have different effects. Although the deep learning can well learn distribution characteristics in sample space, it cannot fully capture the complex factors behind the data because of its weakness of generalization ability [24]. Secondly, although neural network may be good at learning highly complex nonlinear correlations between variables [18], the accuracy of learning result mainly depends on a lot of data for training. A better performance can be got as the amount of data increases [25]. Therefore, it is very important to overcome the shortage of limited data when we use neural network for stock price prediction; otherwise, overfitting in training may happen [26], which finally lead to the weakness of generalization ability.

Some traditional methods can effectively prevent overfitting, such as L1 and L2 regularization, dropout, early stop, reduction of parameters in neural networks, and augmentation of training data [26, 27]. Moreover, additional information is also helpful to mitigate the problem of weak generalization ability. Many researchers gathered textual information from news, social media, and the sentiment of traders in the market and added those information to the prediction model using natural language processing [28–30]. The author in [28] extracted the sentiment information of investors in BBS and combined them with LSTM to predict the market index and sentiment. Mudinas et al. [29] predicted stock price movement using sentiment attitudes and emotions extracted from news. Sardelich and Manandhar [30] investigated the relationship between news and the stock price of the next day as additional information to predict the price of stock. All of those research works have got remarkable achievement in stock price prediction.

Besides additional textual information, more data of a stock in each day is also involved. Reference [19] expanded the inputs of LSTM into the highest price, the lowest price, opening price, closing price, and trading volume. The test on SSEC shows that more features can benefit the prediction of stock price. In [31], Sun et al. used probability function to cluster the stocks which have similar characteristics. A cluster of the stocks is fed into the machine-learning model to predict the trend of a particular stock in the cluster. Experiment result shows that this method can improve the ability of traditional machine-learning model to predict the trend of the stock. In [32], Gao et al. used the technical index of stock price to predict the closing price of the index. The prediction effect of the model is significantly improved. In [33], Baek and Kim proposed ModAugNet-c to prevent overfitting in which a Pearson correlation coefficient is used to choose stocks that are closely related to the stock to be predicted, and then a group of those data is randomly selected as input to expand the training dataset.

On the other hand, as one of the popular data preprocessing techniques, normalization has been widely used in stock price prediction which can reduce noise in the dataset. In [34], Bahanj and Das found that time series forecasting may heavily depend on the normalization technique. In [35], Nguyen et al. denoised stock signals through wavelet transform, so that financial data can be smoothed and get higher accuracy.

In this paper, we propose a model named RLSTM which is based on LSTM and uses a series of random data with uniform distribution against overfitting in forecasting the stock market index. The model contains two modules, that is, prediction module and prevention module. The former acts as the major function in the model, while the latter plays auxiliary role. It is compared with both single LSTM and ModAugNet-c. The results show that its performance outperforms both of them.

The remainder of this paper is organized as follows.  Section 2 introduces LSTM and ModAugNet-c.  Section 3 describes architecture of our proposed model and its training process in detail. Dataset preparation and judgment criteria are also introduced in this section.  Section 4 reveals the experiment results. Conclusions are provided in Section 5.

## 2. Preliminaries

### 2.1. LSTM

LSTM is composed of an input gate, a forget gate, and an output gate. Information can be retained at each time step, so that it could retain longer time-span patterns.

**Figure 1:**
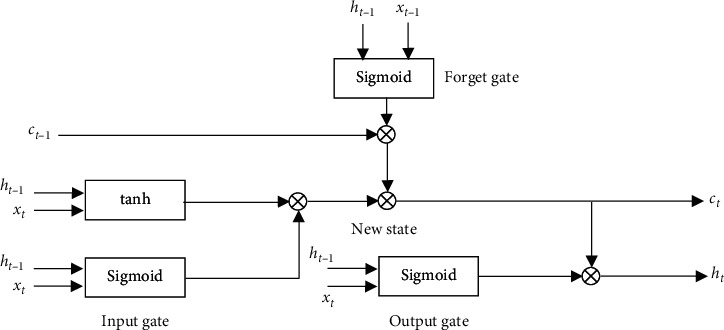
The architecture of LSTM.

Figure 1 shows the architecture of LSTM. Depending on those gates structure, LSTM selectively affects the state of the network at every moment. *x*_*t*_ and *h*_*t*_ represent the input and output states at the moment *t*. The internal formulas of LSTM are shown as follows [36]:

(1) $$\begin{array}{c} \begin{array}{c} f_{t}=\;\sigma \left(W_{f}\cdot \left[h_{t-1},\;x_{t}\right]+b_{f}\right),\\ i_{t}=\;\sigma \left(W_{i}\cdot \;\left[h_{t-1},x_{t}\right]+\;b_{i}\right),\\ \begin{array}{c} \tilde{c_{t}}=\tanh\left(W_{c}\;\cdot \left[h_{t-1},\;x_{t}\right]+b_{c}\right) \end{array},\\ c_{t}\;=\;f_{t}\circ \;c_{t-1}\;+\;i_{t}\circ \;\tilde{c_{t}},\\ o_{t}\;=\;\sigma \left(W_{o}\cdot \left[h_{t-1},\;x_{t}\right]+b_{o}\right),\\ h_{t}=o_{t}\circ \;\tanh\left(c_{t}\right), \end{array} \end{array}$$

where *W*_*x*_ represents the connection parameters between different nodes, *b*_*x*_ represents the bias, tanh is an activation function, and *σ* represents sigmoid activation function.

### 2.2. ModAugNet-c

**Figure 2:**
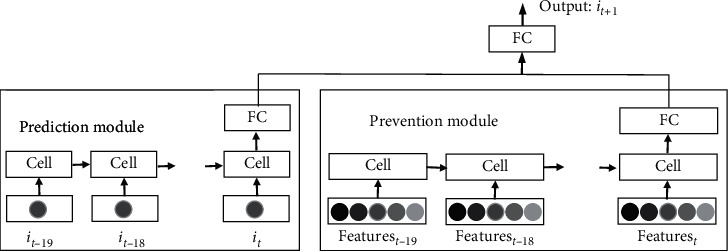
The architecture of ModAugNet-c [33].

ModAugNet-c is a data augmentation framework which consists of two LSTM modules: one acts as overfitting prevention module and the other acts as prediction module [33]. Data of stock market index are input to the prediction module, while 10 other company's stocks that are highly correlated to the stock market index are input to the prevention module for the purpose of preventing overfitting. In training process, 5 of those stocks are taken at a time and fed into the prevention module. There are 252 combinations that can be fed into the prevention module. One of the 252 combinations is chosen in every 200 epochs. The architecture of ModAugNet-c is shown in Figure 2.

## 3. Architecture of RLSTM

### 3.1. Model Architecture

In RLSM, we improve the generalization ability of a single LSTM through adding an additional module which provides random variable, so that the model can show better performance of stock prediction.

**Figure 3:**
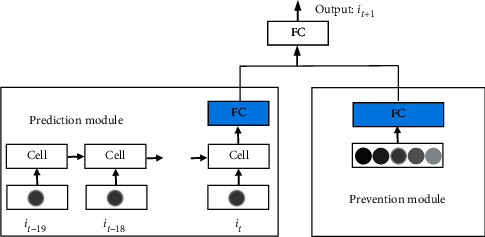
Architecture of the proposed RLSTM.

The architecture of RLSM is shown in Figure 3 which contains two parts. One is prediction module which is composed of a LSTM and a full connection network layer. The input of this module is the prices of the stock we need to predict. The other is prevention module which is only a full connection network layer. Input of the module is random data which are randomly extracted from the uniform distribution within the range between the highest and the lowest prices of a target stock or index. Above the two modules, there is the third full connection layer in the architecture. Its inputs are just the outputs of the two modules. The final result of the architecture can be got after computation of the third full connection layer. Linear activation function is used in the three full connection layers.

We can view the randomness which has no relation with the real stock price as noise in RLSTM. During the training stage, the noise interferes with the model which can help against overfitting for LSTM module.

**Figure 4:**
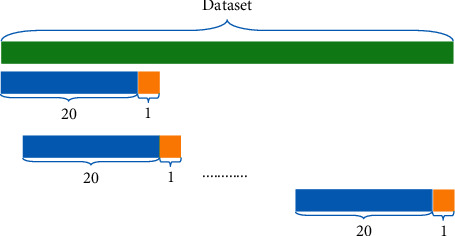
Window slide. The green part at the top is the dataset, and the data of 20 days in the blue part is one time-step data, which is used to predict the data of 1 day ahead at the yellow part.

Depending on the adopted time lag, the network is fed with data within a sliding window and its weights are estimated. In our model, we use prices of successive 20 days (working days of four weeks) to predict one day ahead (short term). For the training dataset we use, a window covering 20 days is taken as input of the model and output predicting price of the 21^st^ day. It is slid in every single day which is shown in Figure 4. The real stock price we need to predict is viewed as label, and the difference between the real stock price and predicting price is obtained as loss and is used for the purpose of backpropagation.

For ModAugNet-c, it requires additional reinforced data to aid the target stock data. The reinforced data are composed of some other stock data which have strong correlation with the target stock data. Such selection of the reinforced data highly depends on personal experience. Different persons may have different selections and thus may result in different performance of training. However, for RLSTM, only the target stock data are required for both training and prediction. The training data for RLSTM are definite and objective which need not be selected from a dataset artificially by experimenters.

The larger the number of layers and weights in deep learning model is, the larger the feature space expressed and the more complex the learning process of the model will be. The time complexity and space complexity will also be larger. Given time series of *N* variables, assume a RLSTM and ModAugNet-c have size *D*, that is, *D* neurons in the layer. In RLSTM, there are 4*D∗D* + 4*N∗D* + 2*D* + 6*D* trainable parameters that lie in the hidden and gate update functions, where 4*D∗D* + 4*N∗D* comes from the transition, 2*∗D* comes from the fully connection layers, and 6*D* corresponds to the bias terms. In ModAugNet-c, there are (4*D∗D* + 4*N∗D*)*∗*2 + 3*D∗*2 + 11*D* trainable parameters that lie in the hidden and gate update function. It is clear that the space and time complexity of RLSTM is smaller compared with ModAugNet-c.

### 3.2. Dataset Preparation and Preprocessing

In this paper, the historical highest daily points of SSEC and S&P500 from January 4, 2000, to September 30, 2019, are used to evaluate the efficiency of RLSTM. Their data points are 4785 and 4967, respectively. These data are publicly available in many financial data providers such as Yahoo Finance and Google Finance.

We normalized the dataset by the following equation to obtain the normalized value $\hat{X}$ at each point [33]:

(2) $$\begin{array}{c} \hat{X}=\frac{X-X_{\text{min}}}{X_{\text{max}}-X_{\text{min}}}, \end{array}$$

where *X* is the original data and *X*_min_ and *X*_max_ are the minimum and the maximum values of the dataset, respectively.

### 3.3. Training Process

Mean square loss is used as loss function in our training progress, while Adam optimizer is used to optimize the model. During training, L2 regularization is adopted to prevent overfitting. Early stop is also used to prevent the overfitting. When the validation loss reaches the minimum, we save the model as the best model.

**Figure 5:**
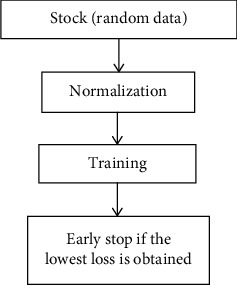
Flow diagram in training.

Figure 5 shows the training process in detail. Both the stock data and the random data are taken as inputs of the proposed RLSTM. The former is input of the prediction module, while the latter is input of the prevention module. Both of them are normalized according to (2) before they are used to train the modules as seen in the second step of Figure 5. Those data used in training stage are split into training and validation dataset for the usage of early stop. An early stop method is used to check the learning curve and stop training before overfitting occurs [37] which is shown in the fourth step of Figure 5. The details of how to split the training data will be explained in section 4. The learning rate is set to 0.00001, the number of hidden units in the prediction module is 32, and batch size is 32.

In the following sections, the first 70% of the dataset is used for training, the following 10% is used for validation, and the remaining 20% of the dataset is used for testing in our experiments.

### 3.4. Judgment Criteria

The following 3 indicators are used to evaluate the results of the test: MSE (Mean Squared Error), MAPE (Mean Absolute Percentage Error), and MAE (Mean Absolute Error). Their formulas are shown as follows:

(3) $$\begin{array}{c} \text{MSE}=\;\frac{1}{N}\sum_{t=1}^{N}{\left(a_{t}-p_{t}\right)}^{2}, \end{array}$$

(4) $$\begin{array}{c} \text{MAPE}=\;\frac{1}{N}\sum_{N}^{1}\left|1-\;\frac{p_{t}}{a_{t}}\right|, \end{array}$$

(5) $$\begin{array}{c} \text{MAE}=\;\frac{1}{N}\sum_{t=1}^{N}\left|a_{t}-p_{t}\right|,\; \end{array}$$

where *a*_*t*_ and *p*_*t*_, respectively, represent the real stock price and the predicted price at time *t*. Those 3 indicators are metrics to evaluate the error of forecasted and actual value, MSE indicates the average squared difference between the forecasted and actual values, and it shows the stability of the model. MAPE measures the prediction deviation proportion in terms of the real value. It is a metric for the forecast accuracy. MAE is the average value of absolute error, which can reflect the actual situation of predicted value error. The lower the value of those indicators is, the better the model performs.

## 4. Experiment

Since the data distribution of each stock has its own characteristics and the noise contained in the data will be different, when different stocks are fed into RLSTM, the number of neurons in each full connection layer needs to be adjusted accordingly. Python language with Pytorch model is used to develop the proposed algorithm. The computing platform uses one GPU card of Tesla P100 to compute gradient.  Table 1 shows the optimal number of neurons in every FC layer.

In Table 2, LSTM output represents the output layer of LSTM in the prediction module. Prediction FC represents full connection layer of the prediction module, while prevention FC represents that of the prevention module. After concatenating output of the two modules together, the last full connection layer is represented by top FC.

In the following sections, if not specifically specified, the first 70% of the dataset is used for training, the following 10% is used for validation, and the remaining 20% of the dataset is used for testing in our experiments.

### 4.1. Performance of RLSTM

The performance of RLSTM is compared with LSTM and ModAugNet-c. The out-of-sample forecasting value are calculated as average 10 times repeated experiments. For example, the forecasting value of repeat experiment *i* (*i* = 1, 2,…, 10) for day t is *a*_*t*,*i*_ and the final experiment result for day t is (1/10)∑_*i*=1_^10^*a*_*i*,*t*_. The three models are evaluated by the judgment criteria shown in (3)–(5), and the corresponding experimental results are shown in Tables 3 and 4, respectively. The bold numbers represent the best values in each set of experiments.

Compared with LSTM and ModAugNet-c, RLSTM gets significant improvement in all the three judgment criteria. As mentioned in section 3.4, it is clear that not only the stability but also the forecast accuracy, RLSTM, has the excellent generalization ability over other models.

**Figure 6:**
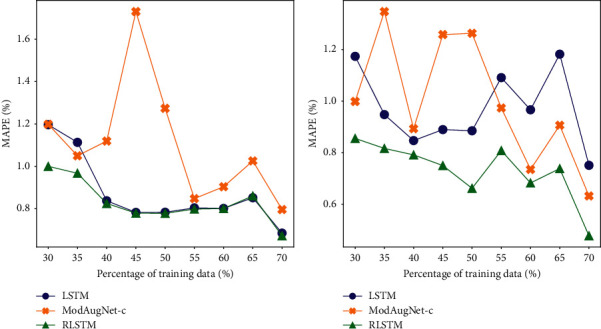
Performance (MAPE) of LSTM, ModAugNet-c and RLSTM under different percentages of training data on (a) SSEC and (b) S&P500.

One of the reasons that lead to overfitting is the inadequate data for training. Generally speaking, the more the training data, the stronger the generalization ability. The MAPE in test under different volume of training data is shown in Figure 6. Here, percentage of training data refers to the ratio of training data in the whole dataset. For example, 30% means the first 30% of the dataset is the training data, the following 10% of the dataset is the validation data, and the remaining 60% of the dataset is the testing data. Basically, as the percentage of training data increases, all the predicting results of those 3 models get better. For SSEC, RLSTM gets the best performance when the percentage increases from low to high. Especially, when it is relatively low, such as at the point of 30%, the performance of RLSTM is significantly better than the other two methods. For S&P500, RLSTM performs better than both LSTM and ModAugNet-c under all of those situations.

In general, when the percentage of training data is relatively small, RLSTM can get the best performance. With the increase of the training data, the predicting results of all those models are almost the same. These experiments show that RLSTM can not only get the best performance in different percentages of training data but also get significant improvement of performance when the training data set is small. When facing the problem of overfitting cased by inadequate training data, RLSTM can still get the best performance.

### 4.2. Analysis of the Trained RLSTM

**Figure 7:**
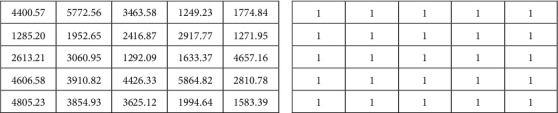
Some fragments of synthetic data. (a) Synthetic data 1 and (b) synthetic data 2.

Synthetic data are generated to investigate how the two modules of trained RLSTM are working to come up with the final prediction. Two types of synthetic data were generated. The first one is called synthetic data 1 whose data are randomly chosen from a uniform distribution ranging within the interval of the highest and the lowest prices of the stock. It is also used as input of the prevention module during the training stage. The second one is called synthetic data 2 whose data are randomly chosen from a fixed value, such as 1.  Figure 7 demonstrates some fragments of these synthetic data as an example.

During the prediction stage, there are three types of datasets used as input of the prediction module. The first one is real stock data, the second one is synthetic data 1, and the third one is synthetic data 2. Two types of datasets are used as input of the prevention module. They are synthetic data 1 and synthetic data 2. Taking into account inputs of both the prediction and the prevention modules, there are 6 different compositions of test datasets shown in Table 5.

**Figure 8:**
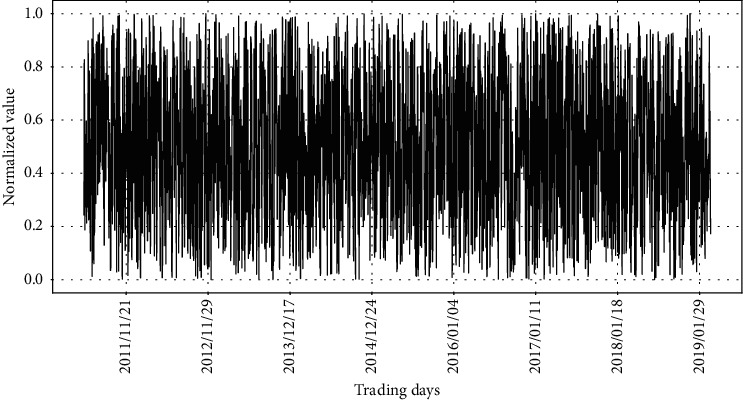
Example of the normalized synthetic data 1.

Note that in the training stage, the real stock price and the synthetic data 1 are used to train RLSTM. After we get a trained RLSTM, the 6 different compositions of test datasets are used in the prediction stage. The length of synthetic data 1 used in the prediction stage is the same as that used in the training stage. Normalization of datasets shown in (2) is necessary before they are input to the model. After normalization, synthetic data 1 ranges within the interval of 0 and 1, which is shown in Figure 8. When module takes synthetic data 1 as input, the output of the module should be like the zigzag patterns, shown in Figure 8. Since the data in synthetic data 2 is a constant value, it becomes 0 after normalization. If synthetic data 2 is fed into the module, the output of the module should be a constant.

#### 4.2.1. Forecasting SSEC

RLSTM is trained by the dataset of SSEC, and the corresponding out-of-sample forecasting errors are summarized in Table 5. MAPE forecasts are taken as a representative and analyzed in detail.

Analyzing from Table 5, it should be noted that the best predicting result can be got when SSEC time-series is fed into the prediction module and the synthetic data 1 is fed into the prevention module. The test MAPE increases slightly as the synthetic data 2 is fed into the prevention module. However, the performance of RLSTM deteriorates dramatically as noised synthetic data are fed into the prediction module. At the same time, we find that the prevention module has only small influence on the final results in the prediction stage. For example, the test MAPE of prediction has only little difference when different inputs are fed into the prevention module and at the same time, SSEC time-series is fed into the prediction module consistently. Such results show that the effect of the prevention module is negligible during prediction stage. The same results can be got when different inputs are fed into the prevention module, and at the same time, the synthetic data are fed into the prediction module consistently.

**Figure 9:**
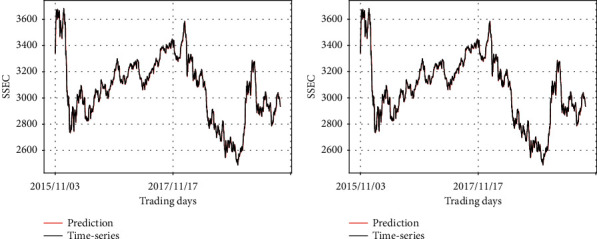
Comparison of the predicting results of RLSTM and the real stock price (SSEC data as the input of the prediction module). (a) Prediction module: SSEC, prevention module: synthetic data 1; (b) prediction module: SSEC, prevention module: synthetic data 2.

**Figure 10:**
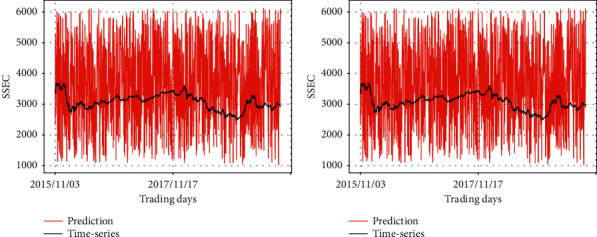
Comparison of the predicting results of RLSTM and the real stock price (synthetic data 1 as the input of the prediction module). (a) Prediction module: synthetic data 1, prevention module: synthetic data 1; (b) prediction module: synthetic data 1, prevention module: synthetic data 2.

**Figure 11:**
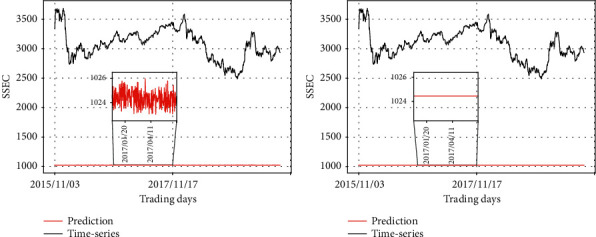
Comparison of the predicting results of RLSTM and the real stock price (synthetic data 2 as the input of the prediction module). (a) Prediction module: synthetic data 2, prevention module: synthetic data 1; (b) prediction module: synthetic data 2, prevention module: synthetic data 2.

Figures 9–11 visually show input-varying test errors. The number above each graph corresponds to the first column of Table 5.  Figure 9 represents the change of predicted values in terms of different data fed into the prevention module while real stock price is fed into the prediction module consistently. The overall pattern of the two graphs reflects the SSEC time-series as input data of the prediction module.

Figures 10 and 11 show the change of predicted values in terms of different input fed into the prevention module, while the synthetic data are always input to the prediction module. It is evident that the prediction module is influential, wherein the shape of its input is mainly depicted in the output graphs. In Figure 10, the two graphs reflect the pattern of the synthetic data 1, which is fed into the prediction module in common. In Figure 11, the two graphs reflect the pattern of the synthetic data 2, which is fed into the prediction module in common.

Remember that the left graph in Figure 9 is the result of the case in which the prediction module takes SSEC as input and the synthetic data 1 is given to the prevention module. Although the synthetic data 1 is still given to the prevention module, if the input of the prediction module changes, we could find a degradation of performance in the left graph of Figures 10 and 11.

At the same time, the graphs also indicate the pattern of the input at the prevention module. For example, the predicting result in the left graph in Figure 11 is basically a straight line, but after local amplification, it can be found that the predicting result shows the zigzag patterns of synthetic data 1, as observed in Figure 8.

#### 4.2.2. Forecasting S&P500

RLSTM is trained by the dataset of S&P500 and the out-of-sample forecast errors are shown in Table 5. The experiments results are similar to that with the dataset of SSEC. When the S&P500 time-series and the synthetic data 1 are used by the prediction and the prevention modules, respectively, the result is the best. If the synthetic data 2 is used instead of the synthetic data 1, the results only become a little worse. As the input of the prevention module has only small influence on the performance, the result is close when the data fed into the prediction module remains the same.

The test MAPE has only little difference when different inputs are fed into the prevention module and the same input is fed into the prediction module. It indicates that the prevention module has only small influence on the final result in the prediction stage.

**Figure 12:**
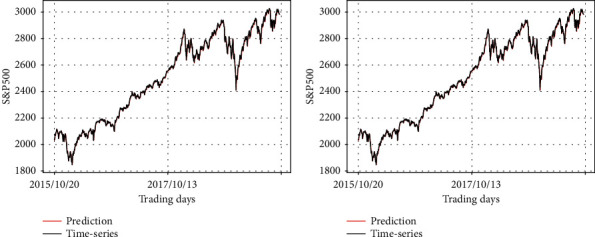
Comparison of the predicting results of RLSTM and the real stock price (S&P500 data as input of the prediction module). (a) Prediction module: S&P500, prevention module: synthetic data 1; (b) prediction module: S&P500, prevention module: synthetic data 2.

**Figure 13:**
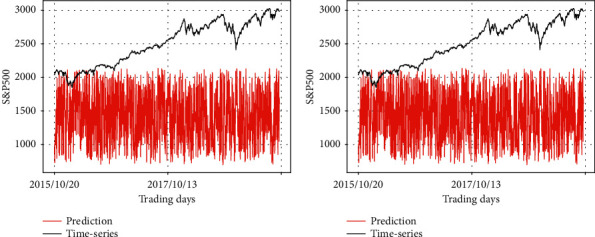
Comparison of the predicting results of RLSTM and the real stock price (synthetic data 1 as input of the prediction module). (a) Prediction module: synthetic data 1, prevention module: synthetic data 1; (b) prediction module: synthetic data 1, prevention module: synthetic data 2.

**Figure 14:**
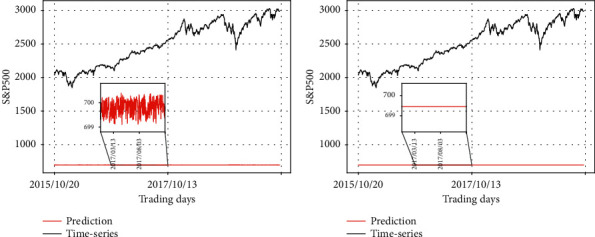
Comparison of the predicting results of RLSTM and the real stock price (synthetic data 2 as input of the prediction module). (a) Prediction module: synthetic data 2, prevention module: synthetic data 1; (b) prediction module: synthetic data 2, prevention module: synthetic data 2.

Figures 12–14 show the test MAPE obtained from the six different test datasets. Note that the number above each graph indicates the first column of Table 6. The change of the predicted values is shown in Figure 12 as different data are fed into the prevention module, while S&P500 is consistently fed into the prediction module. The overall pattern of the two graphs reflects the input data of the prediction module, which is the original S&P500 time-series.

Note that synthetic data 1 at the testing stage is the same as that we fed into the prevention module at the training stage. We did not feed the synthetic data 1 into the prediction module and the synthetic data 2 into any module of RLSTM at the training stage. Thus, it is reasonable that the test performance changes dramatically when these data are fed into the networks. Figures 13 and 14 illustrate the changes of the predicted values as different input data are fed into the prediction module, while the prevention module always takes synthetic data. In Figure 13, we fed the synthetic data 1 into the prediction module in common to produce the two graphs. Note that synthetic data 1 consists of a number randomly chosen from a uniform distribution as depicted earlier in Figure 8. The pattern of synthetic data 1 is highly reflected in the two graphs of Figure 13, although different data are fed into the prevention module.

In Figure 14, the synthetic data 2 is consistently fed into the prediction module. The pattern of it is mainly reflected in the two graphs of Figure 14, despite the marginal difference among the graphs which results from the different data fed into the prevention module. The right graph in Figure 14 comprises the same constant values. Since both modules take the same test data, the constant values are depicted for the network output. After local amplification, the left graph in Figure 14 shows the zigzag patterns of synthetic data 1 as observed in Figure 8 which indicates the input of the prevention module is also reflected in the graphs. But the influence from the prevention module is relatively small.

In this study, we added a prevention module to the overall model to improve the generalization ability of LSTM. The input of the prevention module is a series of random data that are extracted from a uniform distribution, which has no relation with the real stock price of the stock. We can view the input of the prevention module as noise. During the training stage, those noise constantly interferes with the model. The updating of weights is no longer dependent on the fixed relationships, which prevents the network from learning more general features when certain features are only effective under other specific features. From those out-of-sample forecast experiments, we can see that the prediction module plays a major role in the predicting stage, while the prevention module plays an auxiliary role in the same stage. In the testing stage, due to the small impact of the prevention module, the overall model would not depend too much on the information from the prevention module.

### 4.3. Examining Weights of FC Layers

**Figure 15:**
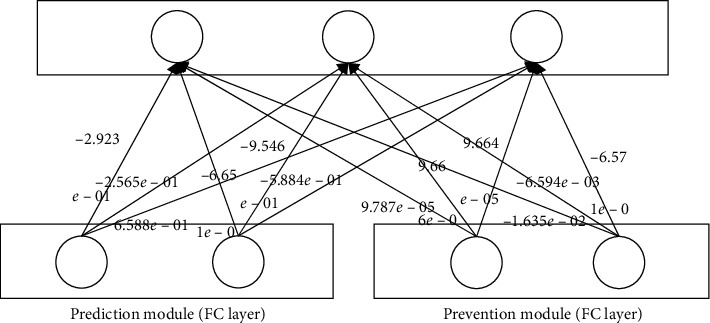
Weights between the fully connected layers of RLSTM to predict SSEC.

**Figure 16:**
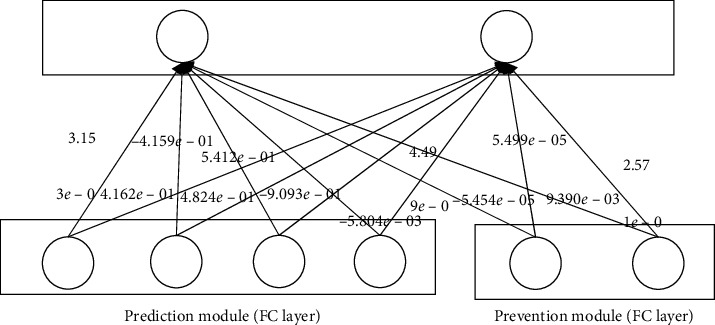
Weights between the fully connected layers of RLSTM to predict S&P500.

In order to further examine function of the prevention module, the weights between the fully connected layers are provided. Exact detail weights in our experiment are shown in Figures 15 and 16. By examining the weights connections from each module to the next layer, we can verify the increased influence of the prediction module after training.

The influence of two modules is provided by calculating the absolute mean values of the two different modules as the magnitude of the weight determines the significance of the node regardless of its sign. They are shown in Table 7.

The absolute mean value of weights from the prediction module is much bigger than that from the prevention module. It means that at the testing stage, the prediction module has much stronger impact on the final results than the prevention module. The latter plays an auxiliary role.

In extracting features based on training data in neural networks, if input variables (the number and order of input variables) are noise which has no meaningful features, it is challenging to learn the features. Therefore, the proposed model, RLSTM, makes the final prediction use only the features learned in the prediction module. The features extracted from the prevention module do not contribute to the final prediction, but they prevent overfitting by causing data augmentation, thus helping to improve generalization accuracy. That is to say, similar to increasing the training data by adding random noise to the original data in the general data augmentation techniques, the prevention module plays a role of amplifying the training data by adding noise [33]. Therefore, in Figures 15 and 16, weights associated with the features of the prevention module are close to zero.

### 4.4. Statistical Tests

In this section, Wilcoxon Signed (WS) rank tests were carried out to verify the equivalence of the prediction accuracy between the two models, which is to show whether there are statistically significant differences between the two-model out-of-sample forecasts [38].

The Wilcoxon signed-rank test examines the null hypothesis that two related paired samples come from the same distribution. In particular, it tests whether the distribution of the difference *x*–*y* is symmetric about zero. It is a nonparametric version of the paired *T*-test. The test is computed using the following steps.

Firstly, subtract the hypothesized mean, u0, from each data value. Rank the values according to their absolute values. Secondly, compute the sum of the positive ranks *S*_*p*_ and the negative ranks *S*_*n*_. The test statistic, *W*_*R*_, is the minimum of *S*_*p*_ and *S*_*n*_. Then, compute the mean and standard deviation of *W*_*R*_ using the following formulas:

(6) $$\begin{array}{c} \begin{array}{c} \mu_{W_{R}}=\frac{n\left(n+1\right)}{4}, \end{array}\\ \sigma_{W_{R}}=\sqrt{\frac{n\left(n+1\right)\left(2n+1\right)}{24}-\frac{\sum t^{3}-\sum t}{48}}, \end{array}$$

where *t* represents the number of times the *i*^th^ value occurs.

Finally, we can compute the *z*-value using

(7) $$\begin{array}{c} z_{W}=\frac{W_{R}-\mu_{W_{R}}}{\sigma_{W_{R}}}. \end{array}$$

The significance of the test statistic is determined by computing the *p* value using the standard normal distribution. If this *p* value is less than a specified level (usually 0.05), the null hypothesis is rejected in favor of the alternative hypothesis. Otherwise, no conclusion can be reached.

The WS test results are shown in Table 8. The value below the diagonal indicates the *p* values for SSEC, and the values above the diagonal indicate the *p* values for S&P500.

The null hypotheses of the WS tests assume an equal level of prediction accuracy between the two models. We reject the null hypothesis under the *p* values less than 0.05 and we can conclude that the prediction accuracy of the two comparative models is significantly different. Otherwise, we cannot reject the null hypothesis at significance levels of 5% or lower if the *p* value is greater than 0.05. In both stock markets, we confirm that the outperformance of our proposed RLSTM is statistically significant.

## 5. Conclusions

In recent years, more and more deep learning methods are used to predict stock price because of the advantages of its nonlinear mapping and strong generalization ability. However, there are many factors that can influence the stock price and the stock price data are nonlinear [23], which makes it hard for the deep learning model to predict its future prices. In addition, deep learning is a data-driven approach, and insufficient data may lead to low accuracy of prediction. To overcome these problems, RLSTM is proposed which is composed of prediction module and prevention module. The prediction module is the major function of the model, while the prevention module is auxiliary. The model is simulated with SSEC and S&P500 dataset. Simulation results show that RLSTM performs better than other modules, especially in the case that the volume of training dataset is relatively small.

This study considered only financial quantified time-series; however, many other factors may influence the movement of stock market, such as politics and psychology of investors [23]. In addition, the proposed network could be trained for the purposes of trading by adding supplementary classifier or regressor, so that the model can additionally output the direction or the expected return of an asset. We only use daily highest price of the stock market index. However, the use of technical indicators will help in making profitable models. Thus, our future task involves effectively dealing with those multimodal data by designing an optimized mutilmodular trading system.

## Figures and Tables

**Table 1:** The parameters in our experiments.

Parameters	Value
Learning rate	0.00001
Number of hidden units in prediction module	32
Batch size	32
Percentage of training dataset	70%
Percentage of validation dataset	10%
Percentage of testing dataset	20%

**Table 2:** The relationship between the stock market index and the optimal number of neurons in every FC layer.

	LSTM output	Prediction FC	Prevention FC	Top FC
SSEC	3	2	2	3
S&P500	2	4	2	2

**Table 3:** Performance of forecasting SSEC.

	MSE	MAPE (%)	MAE
LSTM	970.8654	0.6850	20.8387
ModAugNet-c	1080.5402	0.7962	24.4809
RLSTM	**909.1492**	**0.6724**	**20.5002**

**Table 4:** Performance of forecasting S&P500.

	MSE	MAPE (%)	MAE
LSTM	592.4743	0.7510	19.4220
ModAugNet-c	470.4022	0.6321	16.3951
RLSTM	**286.8698**	**0.4774**	**12.0105**

**Table 5:** Testing results of MAPE of RLSTM on SSEC.

	Prediction module	Prevention module	MAPE (%)
(1)	SSEC	Synthetic data 1	0.6724
(2)	SSEC	Synthetic data 2	0.6728
(3)	Synthetic data 1	Synthetic data 1	44.5287
(4)	Synthetic data 1	Synthetic data 2	44.5291
(5)	Synthetic data 2	Synthetic data 1	66.4628
(6)	Synthetic data 2	Synthetic data 2	66.4598

**Table 6:** Test MAPE of RLSTM on S&P500.

	Prediction module	Prevention module	MAPE (%)
(1)	S&P500	Synthetic data 1	0.4774
(2)	S&P500	Synthetic data 2	0.4801
(3)	Synthetic data 1	Synthetic data 1	42.0591
(4)	Synthetic data 1	Synthetic data 2	42.0713
(5)	Synthetic data 2	Synthetic data 1	71.5464
(6)	Synthetic data 2	Synthetic data 2	71.5590

**Table 7:** Absolute mean of weights of each module to forecast different stock market index.

	Prediction module	Prevention module
SSEC	0.4705	0.0050
S&P500	0.3919	0.0088

**Table 8:** The *p* values of Wilcoxon signed (WS) rank.

	LSTM	ModAugNet-c	RLSTM
LSTM		0.00	0.00
ModAugNet-c	0.00		0.00
RLSTM	0.00	0.00	

The value below the diagonal indicates the *p* values for SSEC, and the values above the diagonal indicate the *p* values for S&P500.

## Data Availability

Data are available from the following link: https://tushare.pro/.
